# Pathogenesis of Metastatic Calcification and Acute Pancreatitis in Adult T-Cell Leukemia under Hypercalcemic State

**DOI:** 10.1155/2012/128617

**Published:** 2011-12-01

**Authors:** Masachika Senba, Kioko Kawai, Naoki Mori

**Affiliations:** ^1^Department of Pathology, Institute of Tropical Medicine, Nagasaki University, Nagasaki 852-8523, Japan; ^2^Department of Pathology, Nagasaki Prefecture Medical Health Operation Group, Isahaya 859-0401, Japan; ^3^Departent of Microbiology and Oncology, Graduate School of Medicine, University of the Ryukyus, 207 Uehara, Nishihara, Okinawa 903-0215, Japan

## Abstract

Human T-cell leukemia virus type-1 (HTLV-1) is the causative agent of adult T-cell leukemia (ATL). Hypercalcemia is common in patients with ATL. These patients rarely develop metastatic calcification and acute pancreatitis. The underlying pathogenesis of this condition is osteoclast hyperactivity with associated overproduction of parathyroid hormone-related protein, which results in hypercalcemia in association with bone demineralization. The discovery of the osteoclast differentiation factor receptor activator of nuclear factor-**κ**B ligand (RANKL), its receptor RANK, and its decoy receptor osteoprotegerin (OPG), enhanced our understanding of the mechanisms of ATL-associated hypercalcemia. Macrophage inflammatory protein-1-**α**, tumor necrosis factor-**α**, interleukin-1, and interleukin-6 are important molecules that enhance the migration and differentiation of osteoclasts and the associated enhanced production of RANKL for osteoblast formation. In this paper, we focus on metastatic calcification and acute pancreatitis in ATL, highlighting recent advances in the understanding of the molecular role of the RANKL/RANK/OPG system including its interaction with various cytokines and calciotropic hormones in the regulation of osteoclastogenesis for bone resorption in hypercalcemic ATL patients.

## 1. Introduction

Adult T-cell leukemia (ATL) was first reported as a new clinical entity in 1977 in Japan [1, 2]. The predominant physical findings are skin involvement, such as erythroderma and nodule formation due to the infiltration of neoplastic cells, lymphadenopathy, and hepatosplenomegaly. The ATL cells are of mature T-helper phenotype and have a characteristic appearance with especially indented or lobulated nuclei. Hypercalcemia is common in patients with ATL, and such patients often show increased numbers of osteoclasts.

A type C retrovirus was isolated from patients with cutaneous T-cell lymphoma by Poiesz and colleagues in 1980 [3]. This virus was later renamed human T-cell leukemia virus type 1 (HTLV-1). In 1981, Hinuma et al. [4] and Yoshida and colleagues [5] reported the isolation of a type C retrovirus named adult T-cell leukemia virus. The two isolates of human leukemia virus, HTLV-1, and adult T-cell leukemia virus, were later confirmed to be the same species of human retrovirus HTLV type I (US isolate) and ATLV (Japanese isolate) [6].

Approximately 16 to 20 million people are infected with HTLV-1 worldwide, and 1 to 5% of the infected individuals develop ATL during their lifetime [7] caused by the transformation of their CD4+ T cells [8]. In Japan, it is estimated that 1.2 million individuals are infected by HTLV-1, and more than 800 new cases of ATL are diagnosed each year [9]. The disease is endemic in southwest Japan, especially Okinawa, Nagasaki, Kagoshima, and Miyazaki, and also in the Caribbean islands, parts of Central Africa, South America, Melanesia, Papua New Guinea, Solomon island, and Australian aborigines [10–12].

HTLV-1 associated myelopathy was recognized in tropical areas independent of that in the Caribbean [13] and Japan [14]. Subsequently, due to its association with HTLV-1, the disease was named HTLV-1 associated myelopathy/tropical spastic paraparesis (HAM/TSP). HAM/TSP is mainly a chronic inflammation of the white matter of the lower thoracic spinal cord, causing spastic paraparesis in the lower limbs [15]. Clinically, HAM/TSP is characterized by higher production of proinflammatory cytokines, such as interferon-*γ* and tumor necrosis factor-*α* (TNF-*α*), and accumulation of Tax-specific CD8+ T cells in the cerebrospinal fluid [16–20].

Patients with ATL frequently develop hypercalcemia. The authors reported four hypercalcemic ATL autopsy cases with metastatic calcifications [21, 22] including one with acute pancreatitis [21]. The reported incidence of acute pancreatitis in the registered ATL cases in Japan is 4% [23, 24]. Patients with ATL are also reported to be positive for parathyroid hormone-related protein (PTHrP) in ATL cells [25]. Furthermore, marked activation of osteoclasts was noted in the bone marrow of these patients, which could be due to the enhanced production of PTHrP in ATL cells [22].

Mechanical stresses and hormonal changes induce bone remodeling throughout the skeletal system, through osteoclastic bone resorption and osteoblastic bone formation [26]. The osteoclasts are multinucleated cells that originate from the monocytes/macrophages [27, 28]. Experimental evidence suggests that ATL cells stimulate the differentiation of hematopoietic precursors into osteoclasts [29]. The activity of osteoclasts is regulated by various cytokines and calciotropic hormones including macrophage inflammatory protein-1-alpha (MIP-1*α*), TNF-*α*, interleukin-1 (IL-1), IL-6, IL-11, macrophage-colony stimulating factors (M-CSF), PTH, PTHrP, 1*α*,25-dihydroxyvitamin D3 (1*α*,25(OH)_2_D_3_), and calcitonin [30–32]. Members of the TNF and TNF-receptor (TNFR) superfamily, receptor activator of nuclear factor-*κ*B ligand (RANKL), receptor activator of nuclear factor-*κ*B (RANK), and osteoprotegerin (OPG) also play a key role in the formation and activation of osteoclasts in conjunction with various cytokines and calciotropic hormones [30, 33, 34].

## 2. Metastatic Calcification

The mechanism of calcification in the viscera is categorized into two groups. Metastatic calcification with hypercalcemia occurs when calcium deposits in previously normal tissue whereas dystrophic calcification occurs in previously damaged tissue. (1) Dystrophic calcification in injured or necrotic tissue in a normal serum calcium level, such as tuberculosis, abscess, and hydatid disease. (2) Metastatic calcification can be divided into malignant and nonmalignant causes. Metastatic calcification in malignancy is reported in parathyroid carcinoma, multiple myeloma, lymphoma, leukemia, hypopharyngeal squamous cell carcinoma, synovial sarcoma, breast carcinoma, and choriocarcinoma. There are many causes of benign visceral metastatic calcification, but chronic renal failure is the most common. Most of the other benign causes are related to calcium balance, such as hypervitaminosis D and hyperparathyroidism [21, 35].

 The mechanism of metastatic calcification is not clear. Metastatic calcification deposition can be influenced by release of excess calcium salts from bone, phosphate concentration, alkaline phosphatase activity, and viscera physicochemical conditions under alkalosis. The Ca_3_(PO_4_)_2_ and CaCO_3_ salts precipitate in tissues that have a favorable physicochemical environment under an alkaline pH condition. The liberated Ca_3_(PO_4_)_2_ and CaCO_3_ salts are transported via the blood in soluble form, which increased delivery and precipitation in tissues with alkalosis. Therefore, it is concluded that calcium salts precipitate in an alkaline environment [35, 36].

 Hypercalcemia is one of the most difficult complication to treat in patients with ATL and a common direct cause of early death. Hypercalcemia is more severe in patients with ATL than that associated with other hematological malignancies [37]. The high frequency of hypercalcemia is the most striking feature of ATL; about 70% of ATL patients have high serum calcium levels during the clinical course of the disease, particularly during the aggressive stage of ATL [38]. Several pathological studies of ATL patients with hypercalcemia have indicated that high serum calcium levels are due to increased number of osteoclasts and accelerated bone resorption. This disease state is characterized by increased osteoclast activity with demineralization of bones and hypercalcemia. We reported previously that serum calcium levels ranged from 15.4 to 19.4 mg/dL (normal range: 8.4 to 10.4 mg/dL) in ATL patients with metastatic calcification [22]. The possibility of metastatic calcification should be considered in ATL patients associated with hypercalcemia who have abnormal shadow by roentgenogram [35, 39]. Other useful diagnostic procedures are imaging with computed tomography (CT) [35], magnetic resonance (MR) [40], and bone scintigraphy [35].

**Figure 1:**
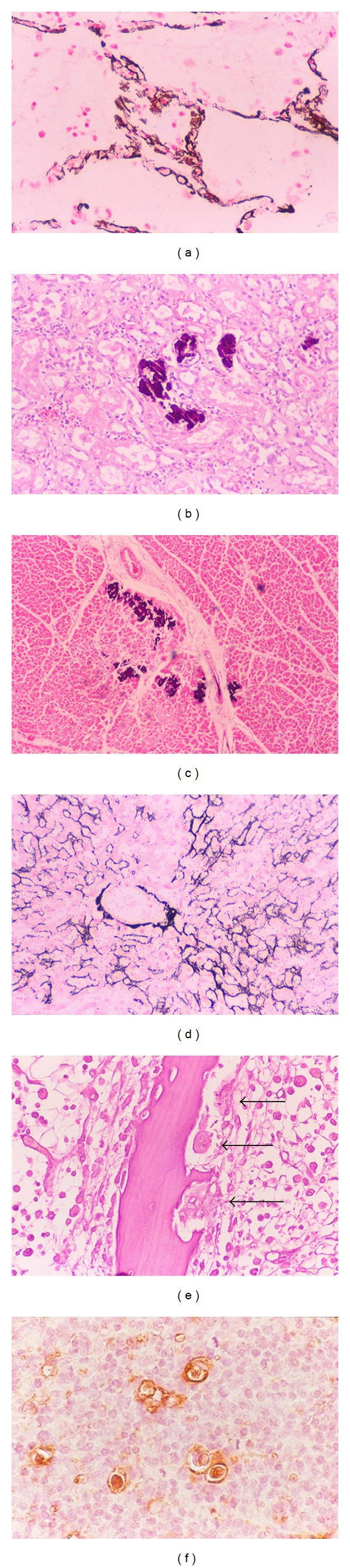
Hypercalcemia in ATL is associated with metastatic calcification. (a)–(d): Metastatic calcification is seen in the pulmonary alveolar septa of the lungs ((a) magnification, ×400), renal tubules of kidneys ((b) magnification, ×200), myocardium ((c) magnification, ×100), and Disses's space, hepatic cell membrane, and central vein wall ((d) magnification, ×200). von Kossa's staining for calcium. (e) Osteoclasts are found in the osseous tissue, and infiltration of numerous leukemic lymphoma cells in the bone marrow of the vertebra. Osteoclasts are multinucleated giant cells. Arrows: typical osteoclasts. Hematoxylin and eosin staining. Magnification, ×400. (f) Immunohistochemistry for PTHrP in leukemic lymphoma cells in ATL. PTHrP-positive cells are stained brown. This case was lymphoma type. The large cells were ATL cells, which were infiltrated in normal lymph nodes. ATL cells produce PTHrP, on the other hand, surrounding normal lymphocytes did not produce PTHrP. Magnification, ×400.

 Metastatic calcification in ATL-hypercalcemia is commonly seen in alveolar septa of the lungs (Figure 1(a)), renal tubules (Figure 1(b)), and myocardium (Figure 1(c)). We reported previously the following rates of metastatic calcification in patients with ATL-hypercalcemia: tubules of kidneys: 100%, pulmonary alveolar septa of lungs: 100%, myocardium: 75%, muscular layer of stomach: 50%, lower portion of the aortic media: 50%, gastric mucosa: 25%, testicular tubules: 25%, and liver: 25% (Figure 1(d)) [22]. Metastatic calcification has also been reported in other organs, including the tongue, pancreas, and spleen [41]. Metastatic calcification of Disse's spaces in the liver of patients with ATL was first reported by Haratake and co-workers in 1985 [42], followed by Senba and colleagues in 1990 [21].

Histopathological examination of osseous tissue sections from ATL patients with hypercalcemia show scattered osteoclasts around the cortex in the vertebrae (Figure 1(e)) [22, 36, 37, 41], although all parathyroid glands were histologically normal [22]. Osteoblast activation is accompanied by osteoclast proliferation.

## 3. Acute Pancreatitis with Hypercalcemia

ATL associated with hypercalcemia and acute pancreatitis was first described in 1984 by Hosokawa et al. [43], followed later by other reports in 1990s [21, 44, 45]. Hypercalcemia is difficult to treat and can be the cause of death in ATL [46, 47]. The relation between hypercalcemia and acute pancreatitis in patients with ATL was suggested based on the observation of pancreatitis in hypercalcemic renal transplant recipients [48]. However, the exact reason linking hypercalcemia and acute pancreatitis in patients with ATL remains to be elucidated. A plausible theory [49] is the following sequence: high serum calcium levels increase calcium levels in pancreatic juice, which result in accelerated calcium-dependent conversion of trypsinogen to trypsin, leading to acute pancreatitis. Another possibility involves the high levels of nephrogenous cyclic adenosine monophosphate [47], which stimulate pancreatic secretion in the extralobular ductal system of the pancreas [50], resulting in acute pancreatitis due to occlusion of the pancreatic duct [23].

## 4. PTHrP and Hypercalcemia

PTHrP is a polypeptide hormone discovered in 1987 and is structurally similar to PTH [51–53]. The aminoterminal peptides of PTHrP have PTH-like actions in osseous and renal tissues by binding to a common receptor for PTH/PTHrP (PTH-1 receptor), resulting in hypercalcemia [54–56]. PTHrP was originally isolated from specific tumors as the humoral hypercalcemia of malignancy [57], and is overexpressed in many types of neoplasms [58]. Several cytokines, such as IL-1 and transforming growth factor-*β* (TGF-*β*), and PTHrP have been implicated in ATL-associated hypercalcemia. Among these factors, PTHrP is considered to stimulate osteoclasts, resulting in increased bone resorption. Moreover, IL-2 increases PTHrP production and secretion in HTLV-1 infected T cells [59, 60]. In addition, PTHrP and IL-6 act synergistically in causing humoral hypercalcemia of malignancy [61, 62]. PTHrP is also overexpressed in ATL cells (Figure 1(f)). The HTLV-1 oncoprotein, Tax is a phosphoprotein localized in the nucleus and acts to transactivate the PTHrP gene in ATL cells and is also involved in the transcription of the PTHrP gene *in vivo* [63, 64]. Furthermore, Tax upregulates PTHrP gene expression *in vitro* and also transactivates the PTHrP promoter [65]. Other studies showed that Tax acts in synergy with Ets-1, AP-1, and AP-2, to increase PTHrP gene transcription [66, 67]. Immunodeficient mice implanted with leukemic cells from patients with ATL exhibited hypercalcemia and overexpressed PTHrP [68]. However, PTHrP cannot directly induce the differentiation of hematopoietic precursor cells to osteoclasts [69]. Furthermore, high serum levels of PTHrP are not always associated with hypercalcemia in patients with ATL, suggesting the involvement of other factors in the development of hypercalcemia [70]. The MET-1/NOD/SCID model demonstrated that RANKL expression correlates with the secretion of PTHrP and IL-6, as well as with hypercalcemia [32]. Therefore, PTHrP is not always the major mediator of hypercalcemia in humoral hypercalcemia of malignancy; rather, the latter involves many other factors.

## 5. Osteoclast Differentiation and Hypercalcemia

**Figure 2:**
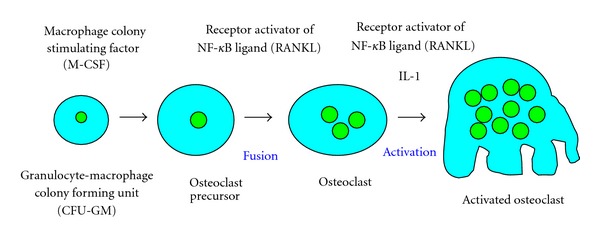
Developmental stages of osteoclast lineage. Osteoclasts are derived from hematopoietic precursor cells, and belong to the monocytes/macrophages lineage. With response to macrophage-colony stimulating factor (M-CSF), hematopoietic stem cells undergo differentiation into the granulocyte macrophage colony forming units (CFU-GM), which are the common precursor cells of granulocytes, macrophages, and osteoclasts. CFU-GM-derived cells differentiate to form mononuclear preosteoclast, which fuse together to subsequently form multinucleated osteoclasts.

**Figure 3:**
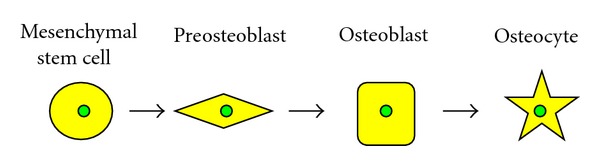
Developmental stages of osteoblast lineage. Osteoblasts are derived from undifferentiated mesenchymal stem cells. The osteoprogenitor cells progress through defined stages from preosteoblasts to osteoblasts and finally to osteocytes, which is responsible of mineralization and calcified bone formation.

Hypercalcemia is one of the most frequent and serious complications in patients with ATL and is due to marked bone resorption associated with osteoclast accumulation. The osseous tissue is consistently remodeled by the bone forming osteoblasts and the bone resorbing osteoclasts. Osteoclasts are multinucleated giant cells present only in the bone. They are derived from hematopoietic precursor cells, and belong to the monocytes/macrophage lineage. Specifically, they are formed mononuclear preosteoclasts, which fuse to form multinucleated osteoclast. The earliest identifiable osteoclast precursor cells are the granulocyte macrophage colony forming units (CFU-GM), which give rise to granulocytes, monocytes, and osteoclasts. CFU-GM derived cells differentiate to committed osteoclast precursors, which are postmitotic cells, and fuse to form multinucleated osteoclasts (Figure 2) [30, 71]. During differentiation of osteoclasts, precursor cells sequentially express c-Fms (M-CSF receptor) followed by RANK [72]. M-CSF and RANKL produced by osteoblasts appear to play an important role in the proliferation and differentiation of osteoclast progenitors [73]. Osteoblasts are derived from undifferentiated mesenchymal stem cells present in the bone marrow, which further differentiate into osteocytes and are embedded in the calcified bone (Figure 3) [74]. The interaction between RANKL and RANK stimulates osteoclast formation and differentiation by activation of several transcription factors that regulate osteoclastogenesis [75, 76].

Molecular biological research has enhanced our understanding of the mechanism of bone resorption. This process is controlled by a system comprised of three key proteins: the RANK, RANKL, and OPG. These proteins mediate bone remodeling and disorders of mineral metabolism in humoral hypercalcemia of malignancy. RANK, RANKL, and OPG are members of the TNF/TNFR superfamily. Several studies have established a consistent relationship between the RANK/RANKL/OPG pathway and skeletal lesions related to disorders of mineral metabolism [29, 30]. The recognition of the RANK/RANKL/OPG system and its interaction with various cytokines and calciotropic hormones in the regulation of osteoclastogenesis have led to further understanding of the mechanism underlying the bone remodeling process in ATL with hypercalcemia.

RANK was discovered by direct sequencing of cDNA from a human bone marrow-derived myeloid dendritic cells [77]. Sequencing of the RANK gene showed it to be a type I transmembrane glycoprotein and also a member of the TNFR family. RKNKL is a TNF-related cytokine expressed by various bone cells including osteoblasts and their immature precursors [78]. Importantly, the target cells of bone resorbing hormones and cytokines are osteoblasts rather than osteoclast progenitors. The expression of RANKL in human and murine osteoblasts is stimulated by various cytokines (IL-1, IL-6, IL-11, MIP-1*α*, and TNF-*α*), and calciotropic hormones including PTH, PTHrP, 1*α*,25(OH)_2_D_3_, and prostaglandin E2 (PGE2) [32, 74, 79, 80]. These are also thought to be important in enhancing the migration and differentiation of osteoclast progenitors into mature osteoclasts by stimulating RANKL production by osteoblasts and stromal cells [30]. PTHrP, IL-11, and PGE2 are most important factors in osteoclast differentiation, which results in RANNKL interaction with the surface of immature osteoblasts [30]. Increased production of RANKL by osteoblasts leads to osteoclast differentiation, resulting in increased bone resorption.

**Figure 4:**
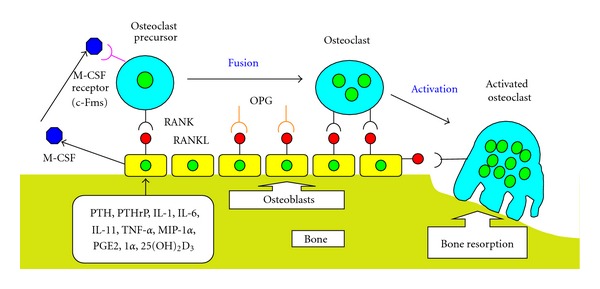
Molecular mechanism of osteoclast differentiation and activation involving the RANKL/RANK/OPG system. Bone remodeling is a balance between formation and resorption through the control of osteoblast and osteoclast activities. Receptor activator of nuclear factor-*κ*B ligand (RANKL), receptor activator of nuclear factor-*κ*B (RANK), and osteoprotegerin (OPG) play important roles in bone remodeling and disorders of mineral metabolism. Bone resorbing factors, such as PTH, PTHrP, IL-1, IL-6, IL-11, and 1*α*,25(OH)_2_D_3_, act on osteoblasts to induce the membrane associated factor called RANKL, which recognizes RANK present on the surface of osteoclast progenitors and osteoclasts. M-CSF is an essential factor for osteoclast proliferation and differentiation, which is produced by osteoblasts in osseous tissue. Reaction of OPG with RANKL inhibits the binding of osteoclast precursors and osteoclasts to RANKL, therefore, OPG acts as a decoy receptor in the RANKL/RANK interaction. Blue structure: M-CSF (macrophage-colony stimulating factor), pink structure: M-CSF receptor (c-Fms), black structure: RANK (receptor activator of nuclear factor-*κ*B), red structure: RANKL (RANK ligand), that is, osteoclast differentiation factor, orange structure: OPG (osteoprotegerin), that is, osteoclastogenesis inhibitory factor.

The decoy receptor OPG with RANKL is also thought to be a key mechanism in the control of bone turnover (Figure 4). OPG was first identified by sequence homology to the TNFR family [81]. OPG is a soluble a glycoprotein secreted by various mesenchymally derived cells such as osteoblasts and bone marrow stromal cells [82]. Reaction of OPG with RANKL inhibits the binding of osteoclast precursors and osteoclasts to RANKL. Therefore, OPG is produced by osteoblasts and acts as a decoy receptor by binding at high affinity to RANKL, therefore, preventing the interaction with RANK [81, 83, 84]. As a consequence of binding to RANKL, OPG acts as an effective inhibitor of osteoclast proliferation, differentiation, activation, and survival, and therefore, it inhibits bone resorption, resulting in bone protection [83]. In this regard, various metabolic regulators modulate OPG expression and secretion by osteoblasts/stromal cells. These include IL-1, TNF-*α*, and TGF-*β*, which increase OPG secretion while various stimulators of bone resorption, such as PTH, PGE2, and 1*α*,25(OH)_2_D_3_ reduce its secretion [78, 79, 85].

## 6. Molecular Mechanisms of Hypercalcemia in ATL

As discussed above, HTLV-1 is the causative factor of ATL, and patients with ATL often exhibit humoral hypercalcemia of malignancy [86], which is induced by PTHrP and cytokines, such as IL-1, IL-6, TGF-*β*, and MIP-1*α* [65, 87–94]. About 70% of ATL patients develop hypercalcemia throughout the clinical course [38]. Overexpression of the RANKL gene correlates with hypercalcemia in ATL. *In vitro* studies have shown that ATL cells obtained from patients with hypercalcemia, which overexpress RANKL gene transcripts, induced the differentiation of human hematopoietic precursor cells into osteoclast in the presence of M-CSF. In contrast, ATL cells from patients with normal serum calcium levels did not induce such differentiation, suggesting that the expression of the RANKL gene in ATL cells is involved in the induction of differentiation of these cells. These results suggest that ATL cells induce the differentiation of the hematopoietic precursor cells to osteoclast through RANKL expressed on their surface, in cooperation with M-CSF, and ultimately cause hypercalcemia [29]. In ATL patients with metastasis and hypercalcemia, activation of the MIP-1*α*, TNF-*α*, IL-1, and IL-6 molecules is induced by Tax-stimulated NF-*κ*B activation [93, 94]. Interestingly, RANKL induces osteoclast formation through the NF-*κ*B signaling pathway, which is critical for osteoclastogenesis. Animals lacking both the p50 and p52 subunits of NF-*κ*B develop severe osteopetrosis [95]. These putative steps in the pathogenesis of disease are supported by evidence derived from tissue culture experiments, xenograft mouse models, and clinical observations in patients [96–100]. Moreover, amino acid sequences homologous to gp46-197 were found in the carboxyl-terminal half of OPG. Administration of the gp46-197 peptide reduced bone mineral density and significantly increased serum calcium levels. The central region of HTLV-1 gp46 acts as an antagonist for OPG and promotes the development of hypercalcemia [101]. HTLV-1 infected cells were found to deregulate the expression of OPG in osteoblast precursors [102]. Ectopic expression of the HTLV-1 basic leucine zipper factor was sufficient to activate Dickkopf-1 transcription in an HTLV-1 infected and uninfected T-cell line [103]. It is possible that HTLV-1 basic leucine zipper factor activates Dickkopf-1 expression at some stage of ATL, thus, indirectly facilitating changes in RANKL and OPG expression, and contributing to the accelerated bone resorption associated with ATL [103].
